# Research on recognition and intervention of behavior sequences in virtual museum learning

**DOI:** 10.1371/journal.pone.0285204

**Published:** 2023-09-05

**Authors:** Xinyi Wu, Xiaohui Chen, Jingwen Zhao, Tingting He, Yongsheng Xie, Chenyang Ma, Wei Wang

**Affiliations:** 1 School of Information Science and Technology, Northeast Normal University, Changchun, Jilin, China; 2 School of Literature and Journalism, Yunnan Minzu University, Kunming, Yunan, China; 3 School of Modern Education Technology, Shenyang University, Shenyang, Liaoning, China; New York University Abu Dhabi, UNITED ARAB EMIRATES

## Abstract

Learning in virtual museum can transcend the limits of time and space. The virtual museum that combines expertise in different disciplines provides a virtual learning environment for college students, but how to intervene in museum learning has been unclear. Targeted at this question, this study selected 2030 majors in clinical medicine from a certain university and the final results exhibited four types of learners who are of high, medium, low and absent museum immersion, respectively. When the learners visited the virtual museum, their behavior data were collected backstage and later used as data source. The method of fuzzy c clustering analysis was utilized to test the behavior recognition results of virtual museum learning, and lag sequential analysis (LSA) was used to carry out sequential transformation of learning behaviors in virtual museum. In this study, the four types of learners were subsumed under two broad categories of middle & high museum immersion and low & absent museum immersion. The importance of behavior was identified with random forest algorithm, and the intervention mechanism of museum teaching was designed according to the analysis results. Specifically, such strategies as museum support, voice guidance, video guidance, sub-museum ordering, rewards points on the list, etc. were used to study the museum learners in need of intervention. The results showed that the learning state of some learners was significantly improved.

## I. Introduction

With the advantages of technology, virtual museums are more widely distributed and less costly than physical museums, and learners have access to them whenever and wherever they want, making it more convenient to serve the needs of informal learning. Martín et al. created virtual museums as an effective learning tool for teaching manufacturing engineering courses [1]. Caroline et al. used virtual museum technology in the design of a language course to enhance the generation and expression of multimodal meaning in student collaboration [2]. The technical implementation and widespread use of virtual museums has also been carried out in China [3, 4], and it has been found that the organic integration of virtual museums with classroom teaching can facilitate students’ thinking development and competence acquisition [5]. It is evident that since the creation and emergence of virtual museums, they have received widespread attention from higher education researchers and are increasingly being used in higher education. However, there are still problems with virtual museum learning such as poor learning outcomes due to learning wandering.

As developed through literature research, research on museum learning behaviour is scarce and focused on physical museum learning. in order to improve the mobile wizard system in museum learning, Sung et al. considered it important to explore the actual patterns of learner access and behaviour in museum learning; to this end, they coded and analysed the learning behaviour of 65 primary school students to identify patterns of behavioural interaction in museum learning, following which derive the characteristics and limitations of the different types of instruction, with higher levels of interaction and more active discussion found in the problem-solving mobile wizard group; and conclude with relevant recommendations for teachers, researchers and instructional system developers [6]. Xu Wei et al. argued that the learning behaviour in museums is called museum learning, where learners have full autonomous control over their learning goals, behaviours and pathways, and provided theoretical and methodological guidance for research on museum learning by elaborating on the museum learning context model and its developmental lineage [7]. Starting from parents’ education and guidance of children, Liang Meirong took children’s museum learning behaviour as the research object, analysed the differences in children’s museum learning behaviour under different family parenting styles, and derived the influence of different parenting styles on children’s museum learning level [8]. Yan Mimi selected young children as the object of the study on museum learning behaviour, analysed the characteristics and influencing factors of young children’s learning behaviour in science and technology museums, and finally put forward rationalised suggestions from both the parent and science and technology museum levels [9].

In summary, this study concludes that research related to virtual museum learning has received some attention and development, with research directions mostly focused on the design and application of virtual museums, with less attention to learner behaviour, especially less research on university students’ professional learning in virtual museums, and even less research on interventions for university students’ museum learning behaviour, and the existing research fails to give, through behavioural analysis The existing studies have failed to provide reasonable solutions to address the problem through behavioural analysis. Therefore, research on learning behaviour in virtual museums needs to be further explored and enriched; whereas research on learners’ online learning behaviour has been more mature, with most researchers carrying out analysis of learning behaviour sequences and construction of models. In addition, it is not difficult to find that lagged sequence analysis is a common method for learning behaviour sequence analysis, which can achieve the identification and mining of learners’ potential learning behaviour [10]. Based on this, this paper proposes to adopt the method of lagged sequence analysis to transform and analyse the learning behaviours of different types of learners based on the learning behaviour identification model of virtual museums, and propose effective intervention strategies based on the characteristics of behaviour patterns and behaviour sequences, in order to improve the learning efficiency of learners and the educational teaching effect of virtual museums.

Based on this this study will use methods such as fuzzy C clustering analysis, lagged sequence analysis and random forest algorithm to identify the importance of museum behaviours to address the problem of poor learning outcomes for low immersion and stray students in virtual museums, aiming to improve the poor learning outcomes in virtual museums. In this study, 2030 clinical medicine learners from a university in northeast China were selected for the experiment, and the behavioural data collected in the background when learners participated in virtual museums were used as the data source, and methods such as cluster analysis with fuzzy C were used to examine the results based on the identification of virtual museum learning behaviours, as well as behavioural transformation of virtual museum learning behaviours based on lagged sequence analysis, resulting in four types of learners. museum high immersion type, museum medium immersion type, museum low immersion type and museum stray type. In this study, the four types of learners were divided into two categories: medium-high immersion type and low stray type, and the behavioural importance was identified through the random forest algorithm. The study then explores how to conduct learning behaviour interventions in virtual museums in order to facilitate learners’ improved learning status and learning outcomes.

## II. Materials and methods

### 1 Research hypothesis

Research Hypothesis 1 The importance of learning behaviour in museums can be identified through the random forest algorithm

Research Hypothesis 2 Effective learning behaviours promote students’ learning outcomes in the museum

### 2 Research methods

In order to prove the hypothesis of this study, experimental research related to virtual museum learning behaviour was carried out using lagged series analysis and data analysis. The lagged series analysis method was used to transform the learning behaviour of the virtual museums to obtain four types of learners: high immersion type, medium immersion type, low immersion type and stray type. The Fuzzy C algorithm and Random Forest algorithm were used for the data analysis method, where Fuzzy C was used to cluster the visiting behaviour of the museum and Random Forest algorithm was used to identify the importance of the behaviour. The intervention mechanism and intervention strategies were designed through the identified important behaviours, so that the intervention effects could be verified whether the important and effective learning behaviours could promote the students’ learning effectiveness in the museum.

### 3 Experimental material: A virtual museum of “Museum of Human Life Sciences”

The experimental material is a virtual museum titled Museum of Human Life Sciences which was designed and developed independently by our research team. In combination with the discipline characteristics of human life science in colleges and universities, the entire learning museum is composed of six sub-museums. Students can learn professional expertise in the museum after login. Before the experiment, the teacher informed students of the total duration of the experiment, that is, 30 minutes, and students were required to complete the learning tasks within the specified time. The technologies applied in museum construction is shown in Table 1, where such tools as VIVE WAVE, Daydream, Unity3d, Virtools were used to develop experimental materials, and technologies of 3D simulation, VR, animation, WebGL, etc. were also integrated to obtain normal learning functions in the experimental virtual museum. The function of backstage data management in the experimental museum makes it possible to identify experimental behaviors. Teachers can view details of student museum behaviour in the museum backend, for example, indicators such as correct answer rate, graphic click completion, and gallery study completion. At the same time some parameters can provide data support for subsequent studies carried out on learning in the museum: visiting terminals, data interfaces, distribution of user IP addresses, etc.

**Table 1:** Technical principle of experimental material “Museum of Human Life Sciences”.

	Technical Parameter
Development Technology	3D Simulation, VR, Animation, WebGL, OpenGL, Unity3D, 3W Engine, Visual Studio, MonoDevelop, SolidWorks, AMESim, Automation Studio, DSHplus, SimulationX.
Development Tools	VIVE WAVE, Daydream, Unity3d, Virtools, Cult3D, Visual Studio, Adobe Flash, VR content display of SDK, etc.
Museum Quality	Total number of single-scene models: 20000 Map resolution: 256*256 Display frame rate: no less than 30 frames per second Refresh rate: 30Hz Normal resolution: ≥1024*768
Museum Backstage Supporter	Development languages: JAVA, Net, PHP, etc.; Development tools: Eclipse, Visual Studio, NetBeans, Baidu VR classroom of SDK, etc.; Databases adopted: HBASE, Mysql, SQL Server, Oracle etc.

## III. Data acquisition and behavior coding of virtual museum learning

### 1 Experimental data acquisition

Selecting the 2030 clinical medicine majors from a medical university in Northeast China, this study collected the backstage data of these students in the experimental museum of “Museum of Human Life Sciences” to investigate their learning behaviors within 30 minutes per round. After eliminating the 30 students whose data were invalid (incorrect registration information, absence from learning, excessive differences in basic knowledge through pretest, etc.), the data set of this study was formed. All students who participated in the virtual museum experiment signed an informational letter that specified the following: the purpose of the study, the process of the study, the significance of the study, and the considerations for participation in the study and post-study remuneration. The students were informed and volunteered to participate in this study.

**Fig 1:**
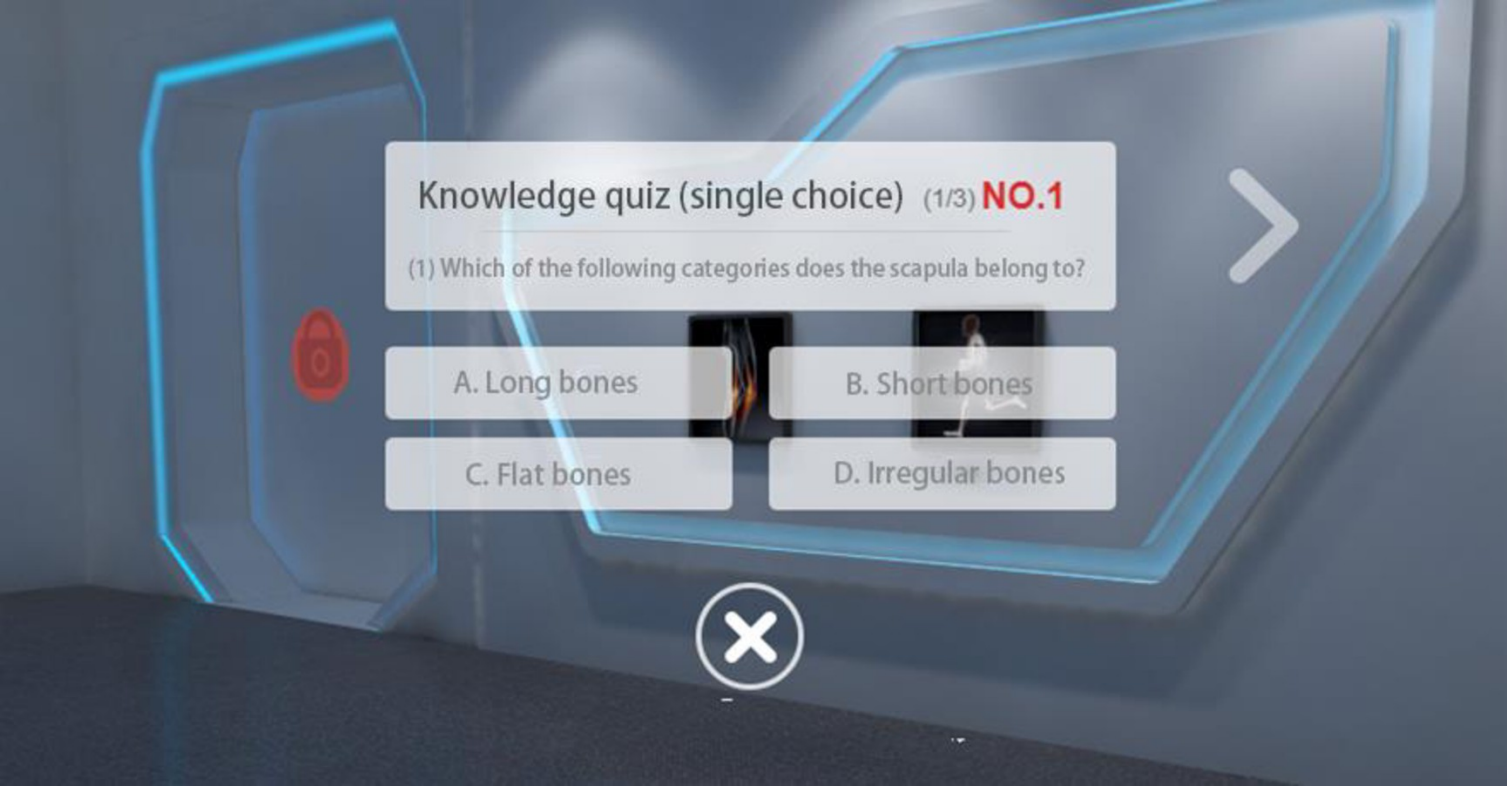
Exhibition of answer test behaviors in experimental virtual “Museum of Human Life Sciences”.

**Fig 2:**
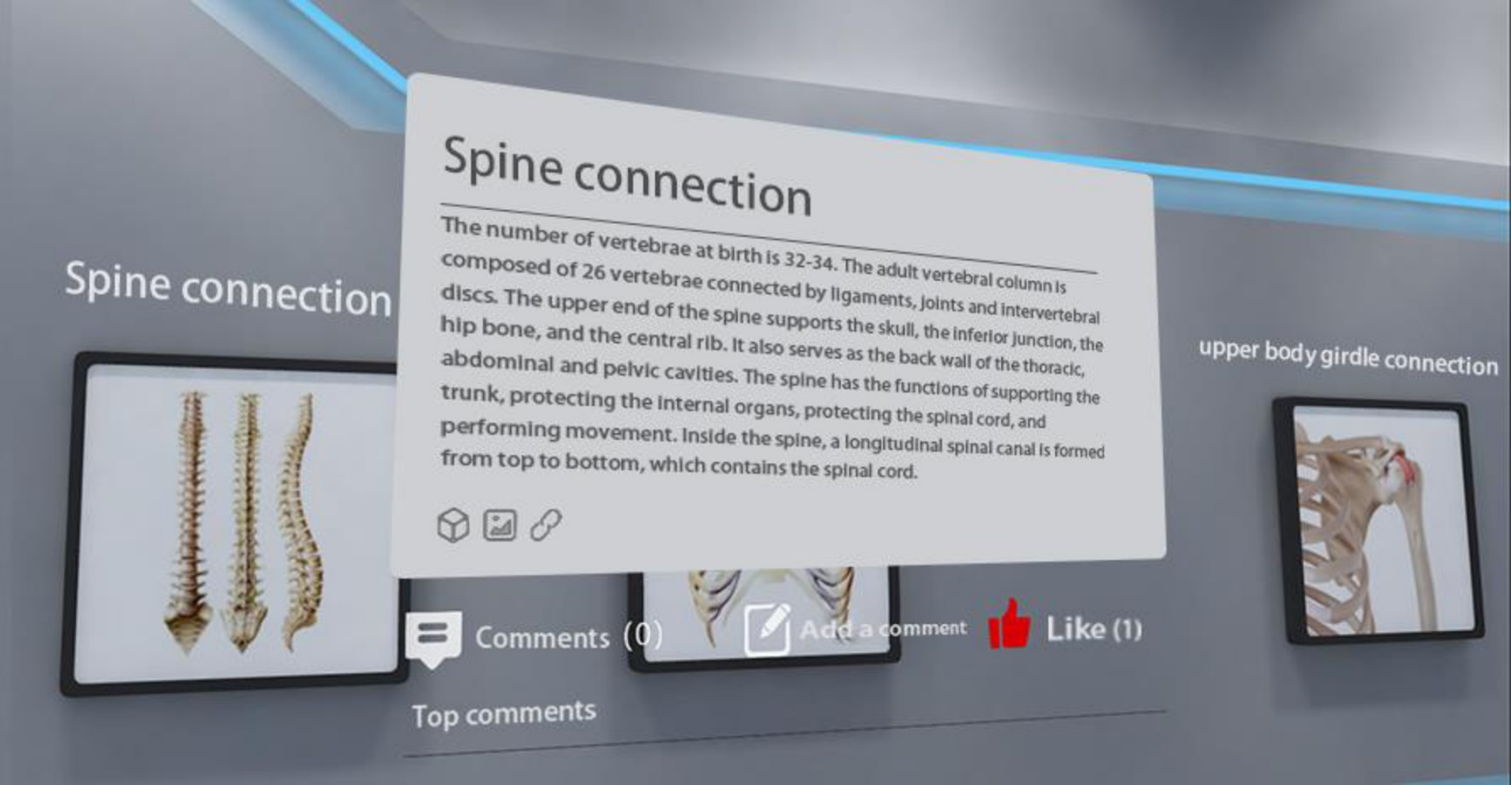
Exhibition of behaviors like comments and thumbs-ups in experimental virtual “Museum of Human Life Sciences”.

Through the backstage supporter of the museum, teachers can fully investigate students’ use traces in the virtual museum and make a statistical analysis of certain basic data. On the museum page, relevant data like daily visitors and the total visitors can be displayed; personal usage data (such as study time, comments, collections, responses, answer, etc.) can be collected and further displayed altogether in the module of “My Museum”. More data can also be collected and docked on the teaching management cloud platform to conduct the statistical analysis of Big Data. Such data include user information (demographic information like sex, education, etc.) and more learners’ behaviors (listening to knowledge, answer tests, discussions, thumbs-ups, comments, browsing content groups, browsing time, use of various functions, etc.). Through the backstage supporter of the museum, teachers can know about students’ learning situation in the virtual museum, so that targeted measures can be put forward to improve teaching. Fig 1 shows the act of answering a test, where students accumulate pass points by choosing the correct answer and can move on to the next sub-court for learning after they have successfully broken through the test; Fig 2 shows the act of liking and commenting related to learning in the museum. The teacher can also use the backend of the museum to understand the students’ learning in the virtual museum, such as attendance rate, course completion rate, correct answer rate, etc., as well as the usage of more users, in order to make targeted improvements and teaching.

### 2 Behavior coding of virtual museum learning

The existing research framework of learners’ behavioral engagement in online and offline learning environments provides a reference for the study of that in virtual museums. In traditional teaching, the research framework of learning behavioral engagement was widely adopted. Li Shuang et al. [11] divided learning behavioral engagement into the six categories of participation, persistence, interaction, concentration, academic challenge and self-monitoring on the basis of the behavioral engagement scale compiled by Lam et al. [12]. With factor analysis, Mou Zhijia et al. [13] split classroom learning behaviors into autonomous, interactive, operational, sensory and teacher-assisted types. In the study of museum learning behaviors, Hou et al. [14] and Sung et al. [15] adopted the “coding scheme of museum learning behaviors” to identify and code learning behaviors, clearly determining the behaviors of museum learners which are visiting exhibitions, recording learning forms, observing exhibits, listening to knowledge, seeking help, partner communication and other irrelevant behaviors.

After analyzing the behavior data transferred from the museum backstage supporter, this study summarized the 13 possible museum learning behaviors of the tested students, which were further classified into 6 types of learning behaviors and non-learning behaviors and coded respectively. The specific coding results are shown in Table 2.

**Table 2:** Behavior coding of virtual museum learning.

Primary Coding	Secondary Coding	Explanation
A(Autonomous learning behaviors)	LK(listen to knowledge)	Students listen to knowledge explained in the museum.
	AT(answer test)	Students gain a pass by completing the answer test.
	SC(self-correction)	Students correct the wrong pass tests.
C(Communicative learning behaviors)	CS(communication & sharing)	Communication between student-student, student-guide
	CC(comment the content)	Students make comments.
	TC(thumbs-up to the content)	Students give a thumbs-up to knowledge cards.
O(Operational learning behavior)	HC(human-computer interactive learning)	Students click on museum pictures and links to expand learning.
S(Sensory learning behaviors)	PM(play music and videos)	Students play music and videos related to museum learning.
	BP(browse pictures)	Students browse the pictures related to learning content.
G(Guided learning behaviors)	RS(record study form)	Students check and complete the study form.
	SH(suggestive help)	Students complete museum content learning according to the suggestive help.
N(Non-learning behaviors)	BM(browse museums)	Students browse the museum aimlessly.
	OT(others)	Other behaviors that have nothing to do with learning.

## IV. Behavior recognition of virtual museum learning

### 1 Classification of learner behaviors based on fuzzy c-means clustering

The method of fuzzy c-means (FCM) clustering is used in this study. In most cases, the objects in a data set can not be separated as distinct clusters since errors are prone to generate if an object is assigned to a specific cluster. In that sense, each object and each cluster should be given a weight value to indicate the extent to which the object belongs to the cluster. Surely, methods based on probability can offer such weight values, but sometimes it is difficult to determine a suitable statistical model. Therefore, the adoption of FCM which boasts natural and non-probabilistic characteristics is a good choice to conduct clustering analysis of the visiting behaviors in the museum. After several iterative processing of the changes in the clustering center, this study divided the browsing behaviors of 2000 visiting students into four categories, as shown in Table 3. The clustering basis is as follows: “browsing→listening”, “browsing→recording study form”, “listening→human-computer interaction”, “listening→answering questions”, “human-computer interaction→commenting”, “answering questions→correction”, “average performance”.

**Table 3:** Clustering analysis of learning behaviors based on fuzzy C.

Behavior Sequence	Clustering 1 (N = 198)	Clustering 2 (N = 487)	Clustering 3 (N = 1109)	Clustering 4 (N = 206)	Total (N = 2000)
BV-LK	231	562	1654	289	684
BV-RS	315	593	2233	457	825
LK-HC	618	916	1209	224	742
LK-AT	636	997	916	136	671
HC-CC	421	639	733	106	475
AT-SC	611	715	827	98	563
	94	86	71	63	76

### 2 Analysis of behavior sequences in different clustering groups

After analyzing the behavior sequences of the four clustering groups, this study has the following discoveries: ①the transformation of behavior sequences in Clustering 1 ranks the highest in type and average times. It means that the visiting students in this group have the best learning effect who in other words, are of the high immersion type of museum learning. Specifically, while visiting the museum, these visiting students show more autonomous learning behaviors and strong subjective initiatives; ②there is a slight difference in the transformation type of behavior sequences between Clustering 2 and Clustering 1, indicating that students in Clustering 2 mainly listen to lectures and answer questions, and seldom make comments. They have relatively good learning effect, belonging to the medium immersion type of museum learning; ③the transformation times of behavior sequences in Clustering 3 is significantly less than that in the first two clustering groups and the transformation is relatively simple. It is worth noting that students in this clustering concentrate more frequently on “browsing the museum→recording form” and “browsing the museum→ listening to knowledge” than those in other clustering, which reveals that students in this clustering acquire knowledge mostly through listening and belong to the low immersion type of museum learning; ④the transformation times of behavior sequences in Clustering 4 is less than that in the previous clustering groups, and most visitors just “browse the museum→ record forms”, suggesting that the visiting students in this clustering only clock in to finish the tasks, lack learning motives and have weak self-control ability, thus belonging to the absent immersion type of museum learning.

Based on the theory of learning behavioral engagement, this study classified the behavior into 4 immersions types, as shown in Table 4.

**Table 4:** Behavior sequences of learners of four different immersion types.

**First: high immersion in museum learning**	human-computer interactive learning→comment and listen to knowledge→answer tests, answer tests→self-correction, self-correction→comment
**Second: medium immersion in museum learning**	listen to knowledge→human-computer interactive learning, listen to knowledge→communication & sharing, self-correction→communication & sharing, answer tests→comment the content, play music and videos→give a thumbs-up
**Third: low immersion in museum learning**	Suggestive help→human-computer interactive learning, browse the museum→human-computer interactive learning, browse the museum→communication & sharing
**Fourth: absent-minded in museum learning**	human-computer interactive learning→others, record study form→others, browse the museum→listen to knowledge, browse the museum→record study form, browse the museum→browse pictures

### 3 Recognition and construction of virtual museum learning behaviors

**Fig 3:**
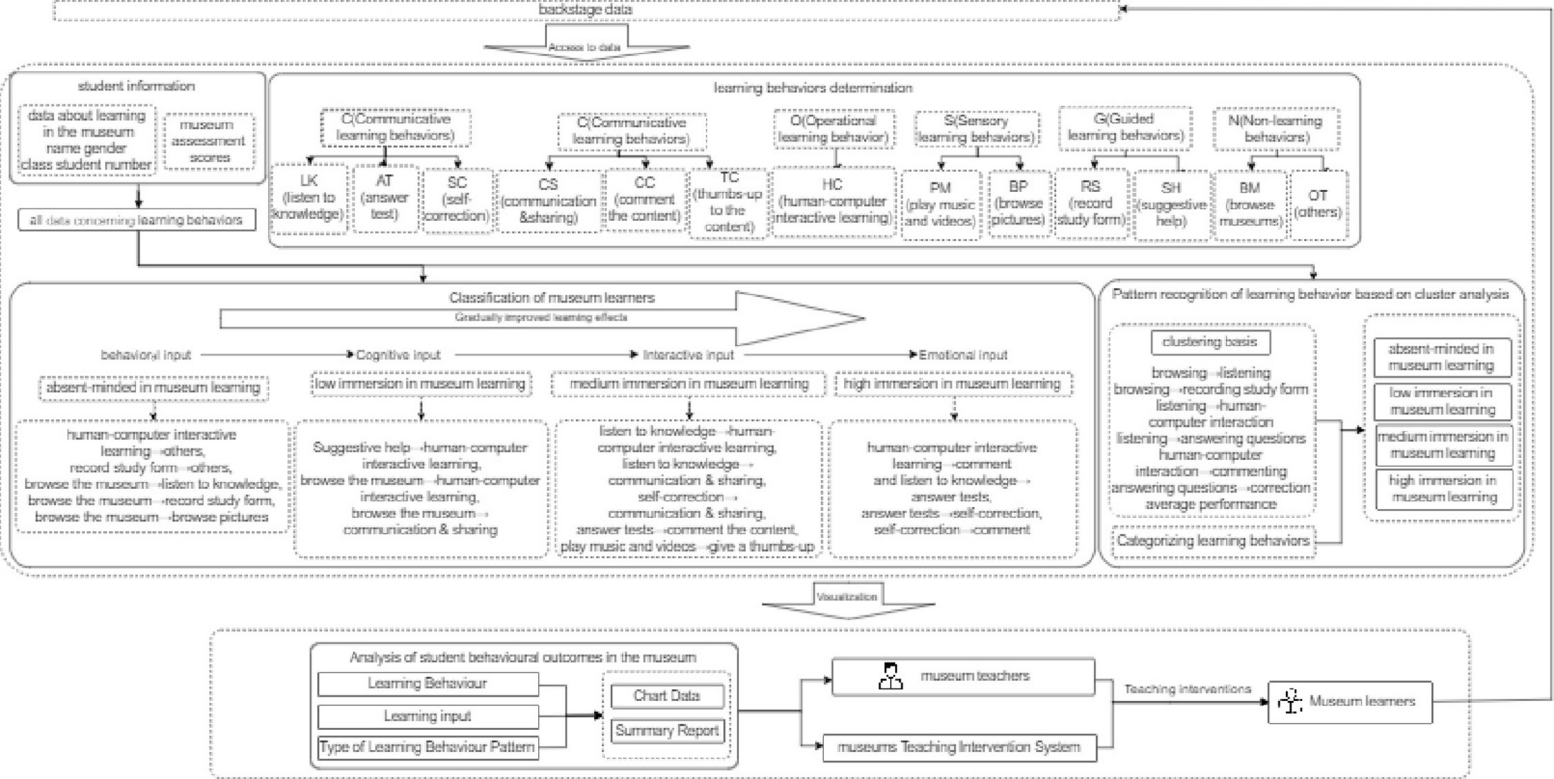
Recognition of virtual museum learning behaviors.

With data processing flow as the analysis framework, this study designed a recognition model of learning behavior patterns (Fig 3) based on museum data using the methods of content analysis, fuzzy c clustering analysis and regression analysis. The model consists of three modules: ①Access to data of museum learning students: the data management function of the virtual museum can record student information and all data about learning in the museum. In the first module of the model, backstage data was transferred, including personal basic information, museum assessment scores and all data concerning learning behaviors. ②According to the behavior sequence record, the learning behaviors transferred in the second module were identified and after analyzing the museum learners, they were divided into the four types of absent, low, medium and high immersion in museum learning. ③After obtaining the analysis report of museum learners’ behaviors and detailed chart data, the teachers intervened in museum teaching through museum teaching intervention system to provide personalized assistance to learners.

## V. Analysis of sequential transformation of virtual museum learning behaviors

### 1 Sequential transformation of virtual museum learning behaviors based on LSA

**Fig 4:**
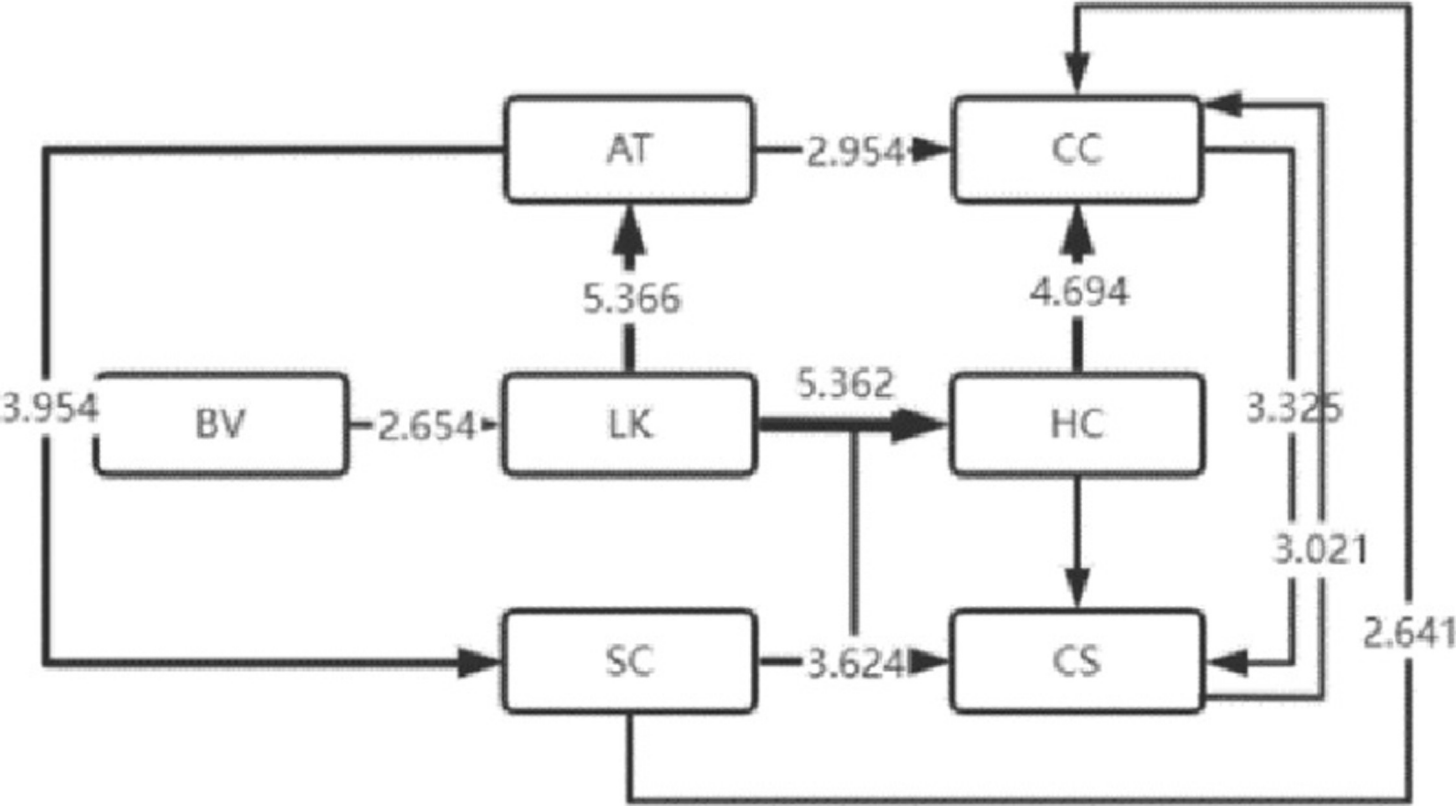
High immersion.

**Fig 5:**
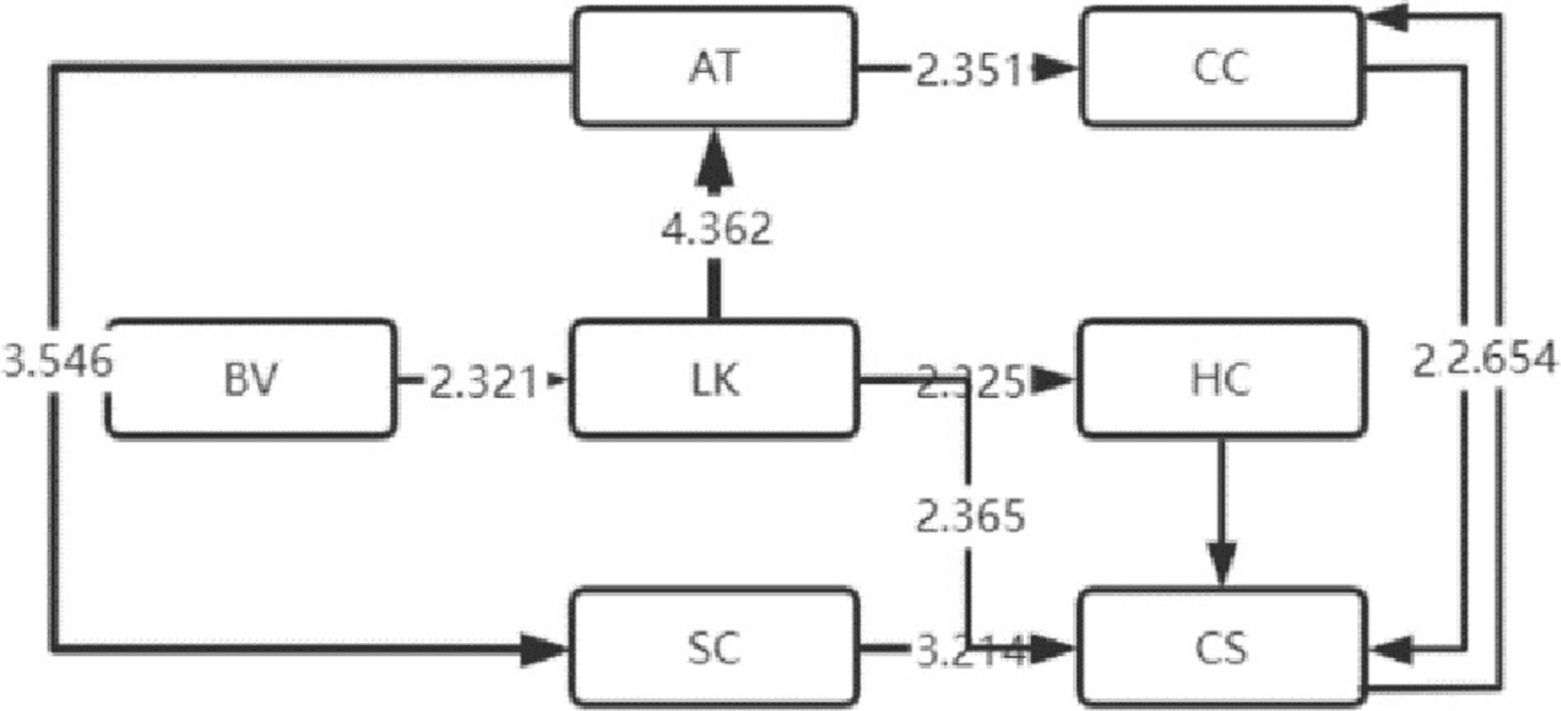
Medium immersion.

**Fig 6:**
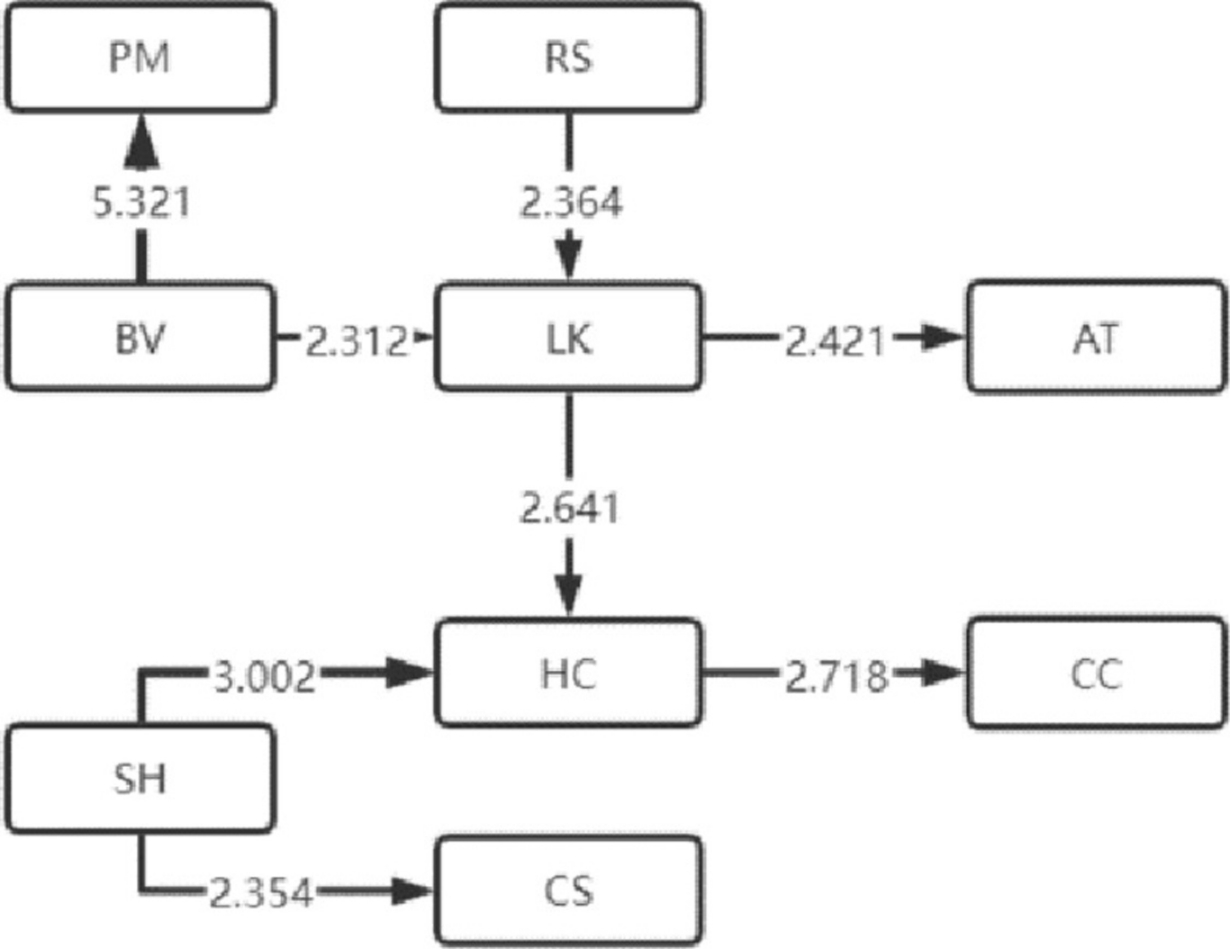
Low immersion.

**Fig 7:**
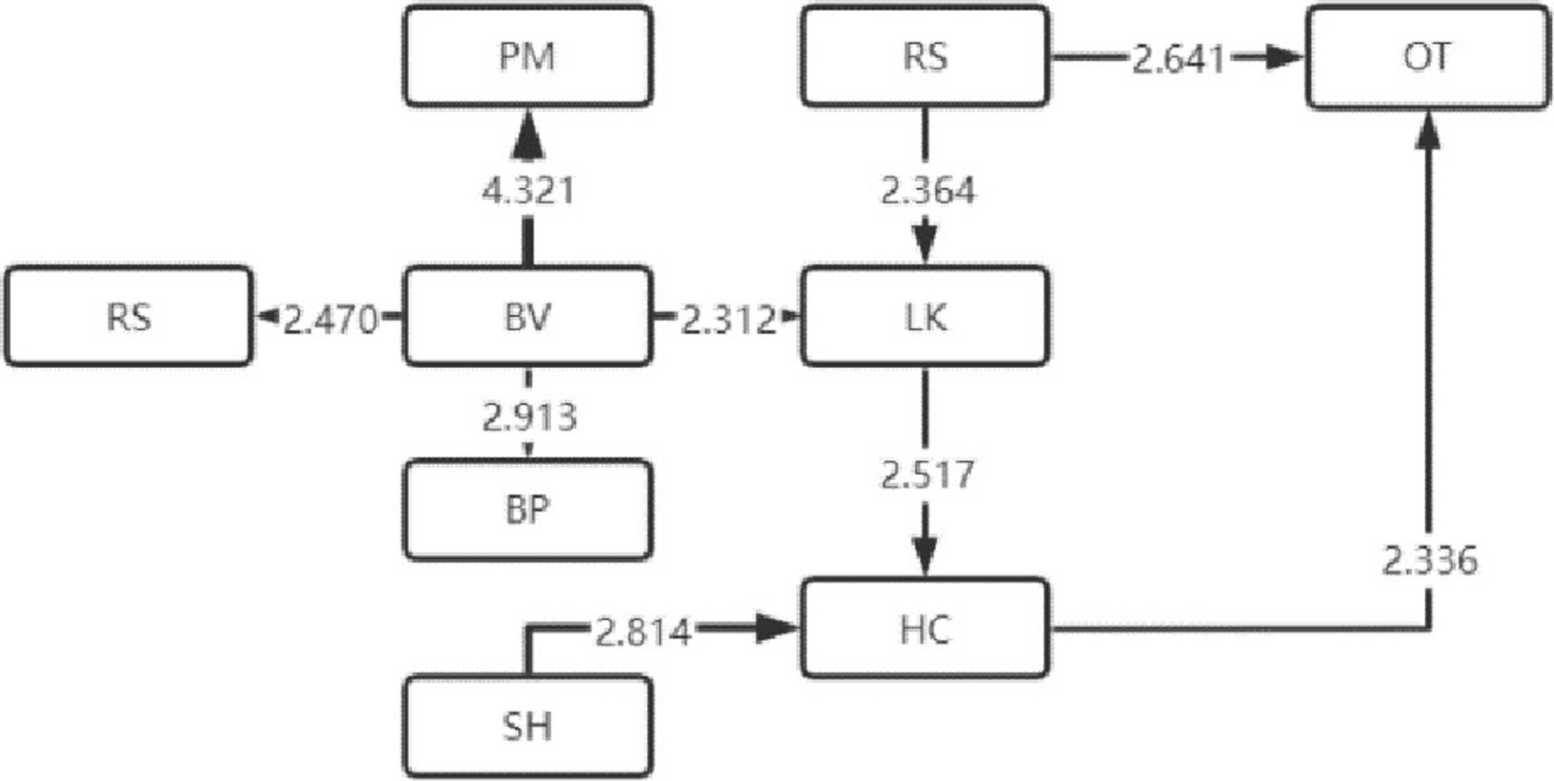
Absent immersion.

The LSA method can lend itself to the sequential transformation of learning behaviors [16]. With this effective analysis method [17], the frequency table of sequential transformation of significant learning behaviors that the four types of visitors exhibited is obtained, based on which, the sequential transformation diagram of their significant visiting behaviors is also drawn, as shown in Figs 4–7. Specifically, arrow direction indicates transformation order, and arrow thickness and number on the line suggest how significant the transformation is (thicker lines and larger numbers represent more significant degrees).

### 2 Analysis of sequential transformation of learning behaviors that visitors of four types exhibit

The sequential transformation of learning behaviors of visitors who belong to the high immersion type in museum learning is continuous, and the length of learning behavior sequence is the longest, indicating that the high-immersion learners performed well in museum learning. The relatively thickest lines for LK-LC represent the greatest salience, i.e. such students are more likely to engage in human-computer interaction learning after listening to the lecture. Meanwhile, the sequential transformation of their learning behaviors is more concentrated and highly structural without independently absent behaviors, suggesting that they have good learning habits.

The length of learning behavior sequence by visitors of medium immersion type in museum learning is the second, demonstrating that the medium-immersion learners have good learning habits. At the same time, significant sequential transformation of independent learning behaviors was manifested in them and no other significant learning behaviors appeared after the sequential transformation of the two learning behaviors of LK (listening to knowledge) and AT (answer test), which indicates that they are more inclined to autonomous learning. Future research will focus on the self-regulated learning of such learners.

The sequential transformation of learning behaviors of visitors belonging to the low immersion type in museum learning is rather scattered compared with that of the first two types of visitors. The sequential transformation of independent learning behaviors was relatively large in number. There appeared no other significant learning behaviors after the sequential transformation of the two learning behaviors of AT (answer test) and CC (comment the content), suggesting that they may just follow the visitors and carry out shallow learning according to the study form. This indicates that such learners are only following shallow learning based on the learning form and that no deep learning phenomena such as problem solving and skill development are taking place.

The sequential transformation of learning behaviors of visitors belonging to the type of absent immersion in museum learning is scattered the most among the four types of visitors. The three behaviors of BM (browsing the museum), PM (playing music and videos) and LK (listening to knowledge) frequently occurred on them, but the rest behaviors were fairly scattered, indicating that they were passive learners’ in museum learning, or to be more straightforward, they were just finishing the learning tasks without deep learning.

### 3 Importance identification and analysis of virtual museum learning behaviors

#### (1) Importance identification of behaviors

**Fig 8:**
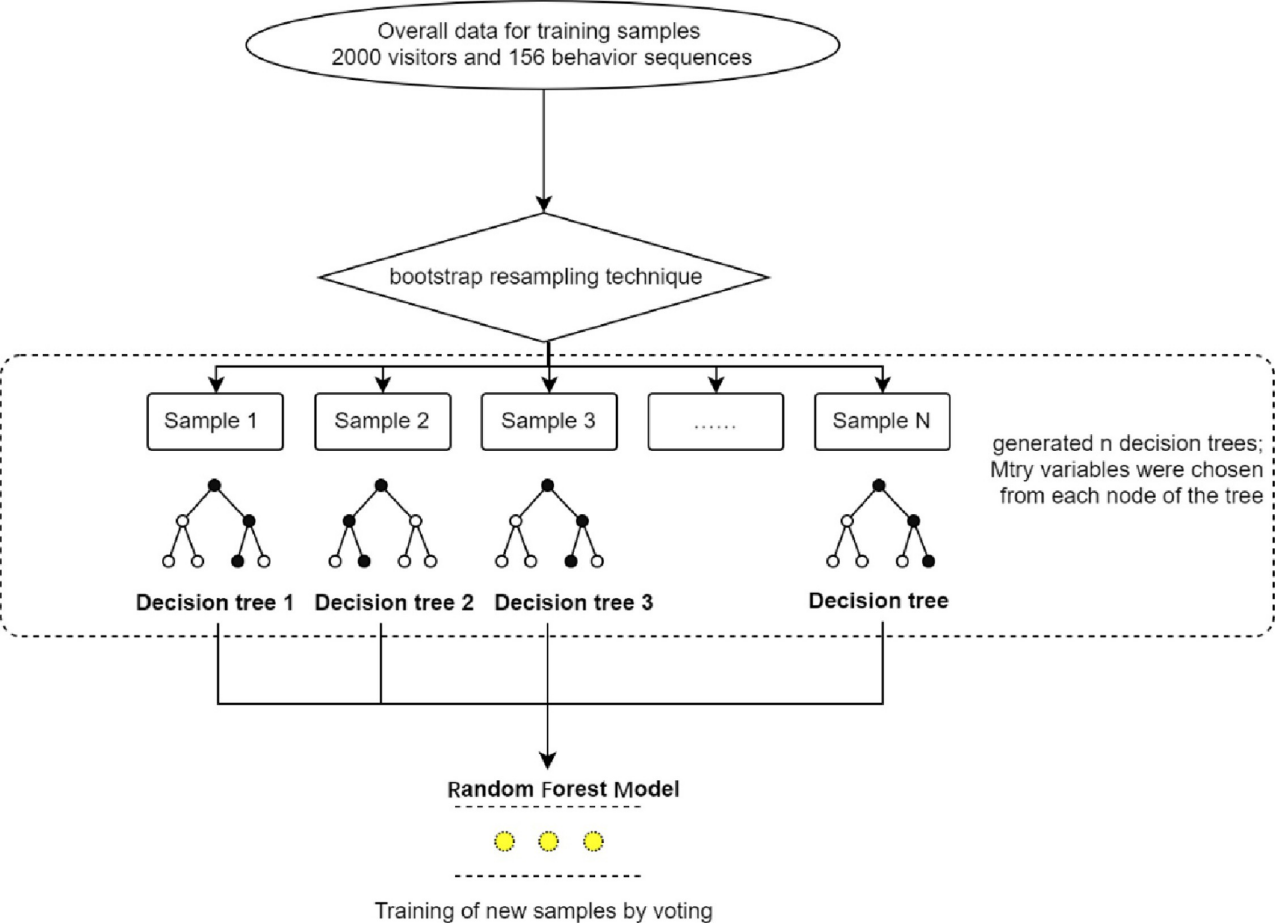
Structural diagram of random forest model.

The combination of the 13 kinds of learning behaviors originating from the 2000 visitors can produce 156 behavior sequences. The 2000 visitors were classified into the four types, among which the high and medium immersion of museum learning were set as 1, and the low and absent immersion of museum learning as 0. The target variables were set as binary variables [18] and the representative random forest (RF) model in the machine learning model was used for analysis [19]. Random forest algorithm boasts fair stability, high tolerance to outliers and noise, and relatively high difficulty to reach over-fitting [20]. In this study, ensemble learning and bootstrap resampling technique were utilized to randomly generate training samples and test samples. As a single training sample generated a decision tree which was a classifier, multiple training samples generated multiple decision trees to form a random forest. The classification of test samples was determined based on the voting scores of the decision trees [21]. The established random forest model is shown in Fig 8.

Finally, to effectively evaluate the performance that the established random forest model exhibited in the diagnosis of students with learning difficulties, the ten-fold cross-verification method was utilized in this study to analyze the accuracy. The four indices of accuracy rate, recall rate, precision rate and F-measure were analyzed with the model, so that a comprehensive evaluation and comparison of the distinction effectiveness based on random forest can be carried out between students with medium or above immersion and those with low or below immersion in museum learning [22]. The binary questions and four cases present in the model form a confusion matrix, as shown in Table 5.

**Table 5:** Table of confusion matrix.

Actual Sample Result	Result Predicted through Model	
	Positive	Negative
Positive	TP (True Positive)	FN (False Negative)
Negative	FP (False Positive)	TN (True Negative)

Accuracy rate: the percentage of correctly predicted results in the total samples. The formula is: Accuracy rate = (TP+TN)/(TP+TN+FP+FN).

Precision rate: the probability of actually positive samples in all samples predicted to be positive. The formula is: Precision rate = TP/(TP+FP).

Recall rate: the probability of samples predicted to be positive in the actually positive samples. The formula is: Recall rate = TP/(TP+FN). The higher recall represents higher probability that actually negative users can be predicted.

TP: The sample was retrieved positive and it was actually positive. (correct identification)

FP: The sample was retrieved positive, but it was actually negative. (primary wrong identification)

FN: The sample was not retrieved positive, but it was actually positive. (secondary wrong identification)

TN: The sample was not retrieved positive, and it was actually negative. (correct identification)

The data were distributed to the training group and verification group in the proportion of 7 to 3. The model training was conducted through the data in the training group, and the verification set was verified with the model after training. The result data of the confusion matrix is shown in Table 6.

**Table 6:** Confusion matrix.

Actual Sample Result	Result Predicted through Model	
	Positive	Negative
Positive	177	29
Negative	50	345

According to the analysis of the confusion matrix which is about the model verification results in the above table, it is found that in the verification samples, there are 206 samples of actually medium and high immersion, of which 177 samples are correctly predicted; there are 395 samples of low and below immersion, of which 345 are correctly predicted. Based on the confusion matrix, the evaluation indicators of the model are calculated, as shown in Table 7.

**Table 7:** Model evaluation.

accuracy	precision	recall	f1
86.9%	78.0%	86.1%	82.60%

That the recall and precision rates in the above table are 86.1% and 78.0% respectively indicates that the identification effect of the model is so satisfactory that it can effectively identify medium and high immersion students and students of low immersion level.

#### (2) Regression analysis

To explore the relationship between learners’ behavior sequences and their academic performance, this study analyzes the importance degree of the variables in random forest model. According to the importance data, the 17 behavior sequences of superior importance are selected, as shown in the following table. The larger importance number signals closer correlation between the two variables, indicating that all the behavior sequences in Table 8 are closely correlated with academic performance.

**Table 8:** Statistical table of field importance.

Sequence Coding of Behavior Classification	Coding of Behavior Sequence	Ranking of Field Importance
A-A	AT-SC	0.1572
A-C	SC-CC	0.1129
O-C	HC-CC	0.1061
A-A	LK-AT	0.0949
A-C	AT-CC	0.0924
O-N	HC-OT	0.0824
A-C	SC-CS	0.0812
A-O	LK-HC	0.0812
A-C	LK-CS	0.0811
G-O	SH-HC	0.0797
N-O	BV-HC	0.0766
S-C	PM-TC	0.0750
N-C	BV-CS	0.0744
G-N	RS-OT	0.0720
N-S	BV-BP	0.0719
N-G	BV-RS	0.0691
N-A	BV-LK	0.0689

Intervention in all behaviors are rather likely to make learners weary of learning, so when teachers consider intervening, the behaviors that highly influence academic performance should be sifted out [23]. According to the above analysis, the corresponding intervention measures are designed in accord with the importance ranking in Table 8. When such behaviors as AT-SC (answer test-self-correction), SC-CC (self-correction-commenting the content), HC-CC (human-computer interactive learning-commenting the content), LK-AT (listen to knowledge-answer test) and AT-CC (answer test-commenting the content) are insufficient, intervention in museum teaching is needed. Specifically, in terms of AT-SC behaviors, hints for answer tests should promptly intervene to make students finish their questions with dispatch; regarding SC-CC behaviors, hints of pictures and texts should promptly intervene to impel learners to think and hand in comments, etc.; hints of pictures, texts and hyperlinks should intervene in HC-CC behaviors; audio guidance can make a difference in LK-AT and AT-CC behaviors insomuch that learners can be motivated to better complete the learning tasks.

When such behaviors as PM-TC (playing music and videos-giving thumbs-ups), BM-CS (browsing the museum-communication & sharing), RS-OT (recording study form-other behaviors), BM-BP (browsing the museum-browsing pictures), BM-RS (browsing the museum-recording study form) and BM-LK (browsing the museum-listening to knowledge) are way too many, timely intervention in museum teaching is also necessary. In the above-mentioned key behaviors, more behaviors of browsing the museum and those have nothing to do with learning tasks occupy students’ time for completing learning tasks. The emergence of other behaviors indicates that learners are liable to low immersion and absent-mindedness in museum learning, so intervention in museum teaching should be launched as soon as possible.

## VI. Design of teaching intervention mechanism for virtual museum learning

### 1 Design of intervention mechanism in virtual museum

**Fig 9:**
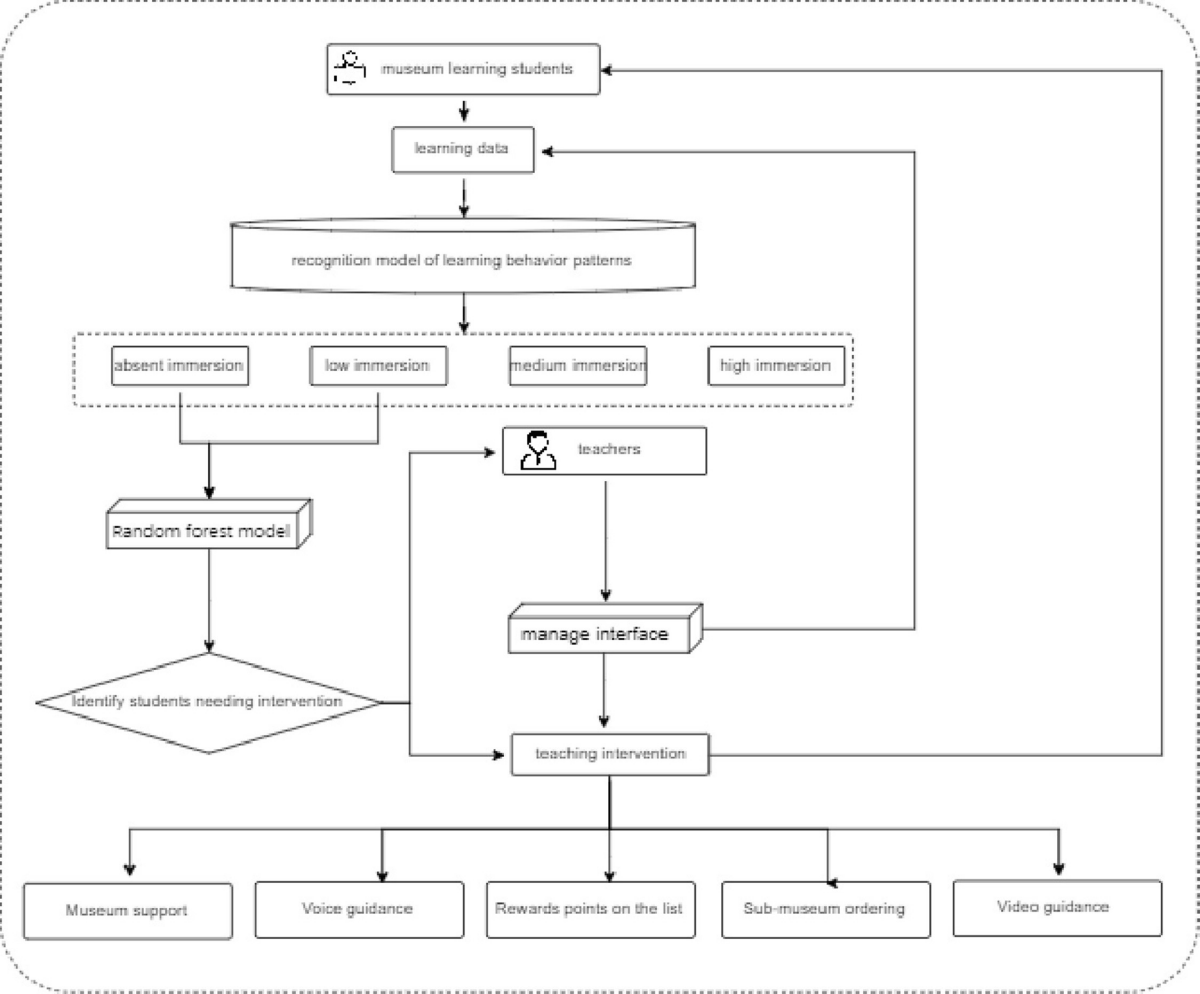
Teaching intervention mechanism.

After analyzing the sequential transformation of learning behaviors of four different types of learners, this study finds out that except for learners of high immersion type, learners of the other three types have certain problems regarding learning and those of the types of absent and low museum immersion especially need teaching intervention. Hence, centered on the museum data-based recognition model of learning behavior patterns, this study has designed a teaching intervention mechanism based on problematic learning behaviors, as shown in Fig 9.

When museum learners are grouped into high, medium, low and absent immersion types, the mechanism quickly determines and identifies the learners who need intervention. The teachers take teaching intervention measures, including voice prompts, help manuals, peer assistance, teachers’ guidance and others, to help them with learning so that they can concentrate and understand the museum knowledge more easily, thereby improving learning effect.

### 2 Design of intervention strategies

This study designed the following virtual museum intervention strategies based on the behaviors in need of key intervention and the characteristics of virtual museum learning tasks: (1) Museum support: dominated by texts, images and hyperlinks, the first two of which were intended to be triggered by learners who therefore, could be stimulated to discover and actively solve problems; hyperlinks were mainly linked to the museum learning manuals or help manuals on other websites. With museum support, learners can complete the tasks with more clear hints. (2) Voice guidance: a countdown was designed in every virtual museum learning task. Specifically, if operational instructions from learners were not received when time was due, the voice guidance prompts would be started automatically, and a voice guidance button was provided for students in the intervention experiment, so they could also turn on voice guidance in advance. The content of voice guidance corresponded to the hints for learners’ current operating steps, contributing to their understanding of task flows and solutions. (3) Sub-museum ordering: learners were allowed to preview the themes and difficulty degrees of the 6 sub-museums in advance (difficulty degrees: simple, medium, and high difficulty), and willingly chose the appearance order of sub-museums before entering the intervention experiment. The familiar answering habits made for their more positive performance in the museums. (4) Rewards points on the list: the strategy of rewards points can lend itself to the intervention of all important behaviors. Answer tests were designed at the connecting door between two sub-museums and the tests were about expertise of the current sub-museum. The strategy of rewards points on the list was used as an incentive measure and every successful pass of the test would be displayed in the eye-catching position of the virtual museum that “Your Rewards Points Have Been Accumulated on the List”. Positive incentive of rewards points would motivate learners to study, further promoting the completion of museum learning tasks. (5) Video guidance: when museum support and voice guidance failed to be commonly accepted by learners, video guidance could be started autonomously. The content of video guidance was displayed in the form of pop-up videos, where the teachers, acting as operators, interpreted the operation steps of the current task, thus assisting learners with their learning tasks more intuitively. That video guidance was last activated among all the strategies since it was the most direct intervention strategy could impel learners to autonomous learning.

### 3 Effect analysis of teaching intervention in virtual museum

Psychologist Albert Bandura believed that learning is the imitation of specific peer behaviors [24]. By observing peer behaviors, learners substitute and reinforce the corresponding behaviors [25]. If a certain peer behavior is positively recognized by society, learners are also more liable to this behavior [26]. In other words, the continuous enhancement of a peer behavior also predicts successful results for learners, and vice versa. Therefore, based on the above research results and the concept of imitation example, this study intervenes in the study of learners’ with poor academic performance to explore the guiding effect that the learning behavior patterns of learners with fair academic performance exert on those with relatively poor performance [27].

**Fig 10:**
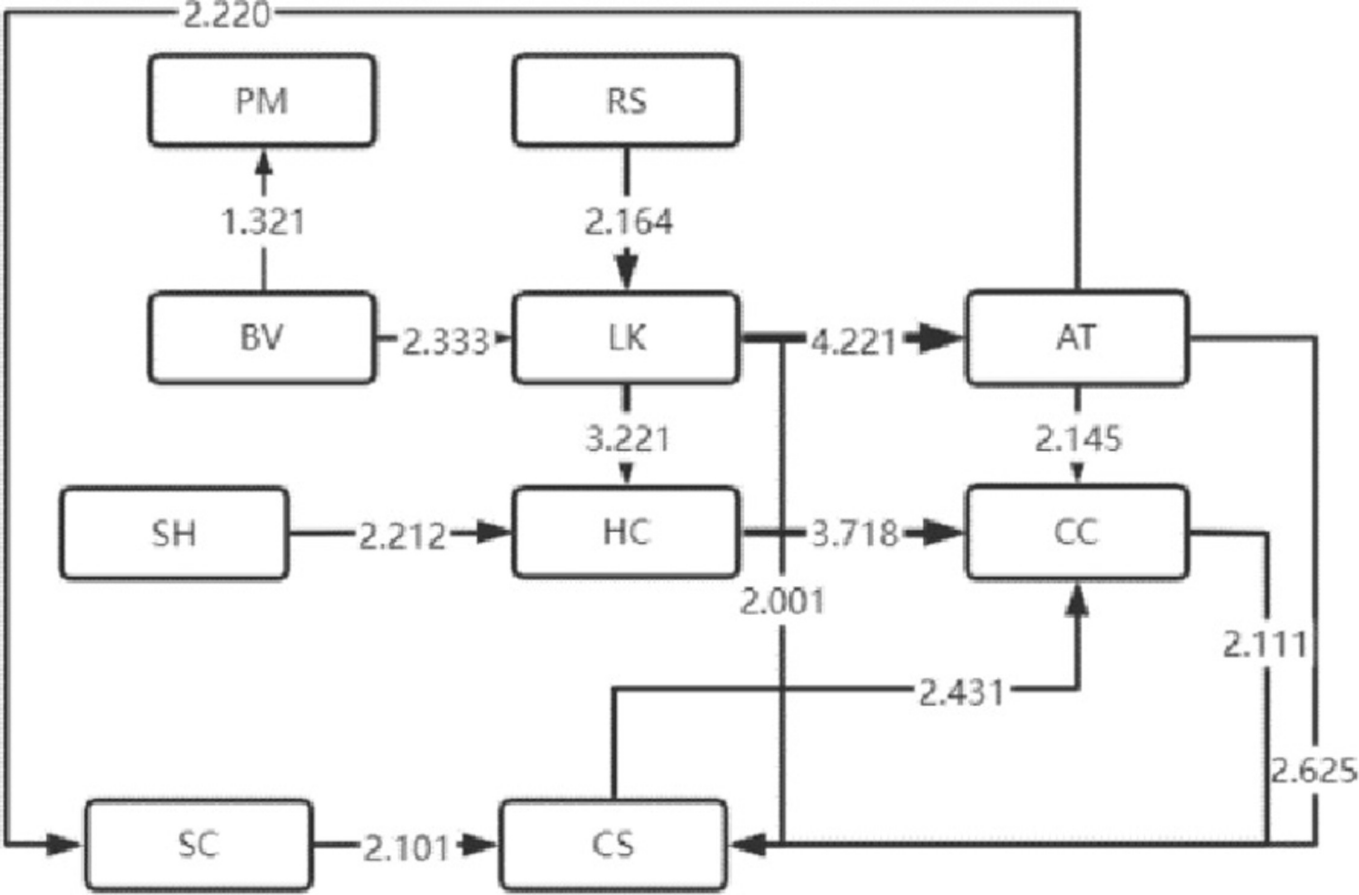
Improvement of low-immersion learners.

**Fig 11:**
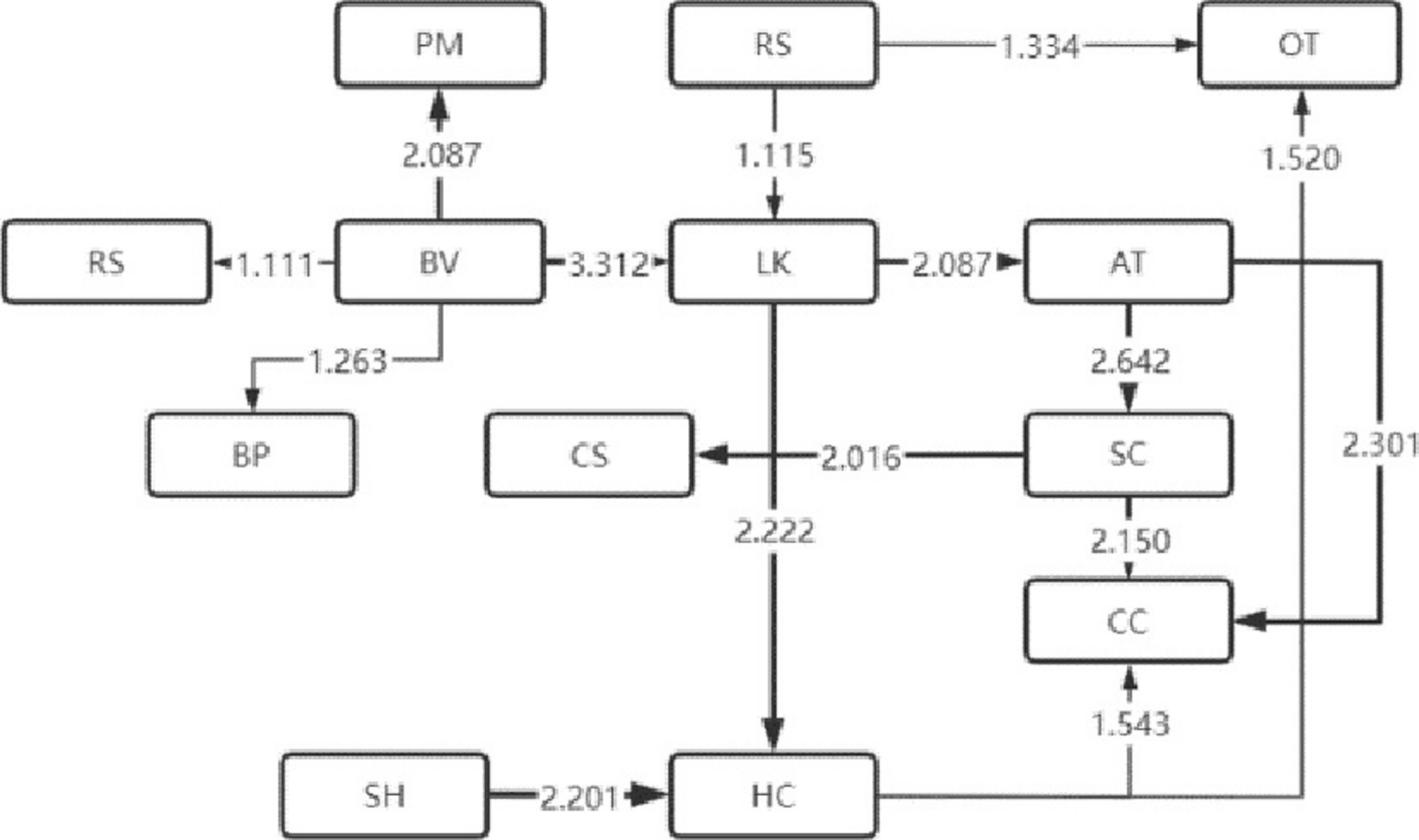
Improvement of absent-immersion learners.

1. Intervention subjects: learners of the absent and low museum immersion after the first round of experiment. The museum learning materials which were of the same layout and difficulty degrees as the “Museum of Human Life Sciences” in the first experiment were used to do the intervention experiment. The duration of the experiment was 30 minutes.
2. The intervention result analysis is shown in Figs 10 and 11: comparative analyses of the results are demonstrated in Figs 10 and 11. Among the 1,109 learners who were of the low immersion type, after intervention, 165 of them reached high immersion, 768 of them medium immersion, and the rest remained unchanged. Among the 206 learners who were of the absent immersion type, after intervention, 20 of them reached high immersion, 97 of them medium immersion, 67 of them low immersion, and 22 of them remained absent immersion type. The above results reveal the difference that intervention has made in improving some learners’ learning status. Therefore, in future museum teaching, teachers should actively intervene to make students improve their learning behaviors, thereby making a difference in the improvement of students’’ learning effect.

## VII. Results and discussion

This study verifies hypothesis 1: the Random Forest algorithm can identify the importance of the learning behaviour of the museum. After extracting the learning behaviour data of virtual museum learning, a scientific method is needed to cluster and identify the importance of the behaviour, so this research innovation uses the fuzzy C clustering algorithm and the random forest algorithm after experimental verification, the random forest algorithm is stable and tolerant to outliers and noise, to derive the importance of the behaviour and based on the analysis to derive the museum teaching intervention mechanism. The research hypothesis 2 was also verified: effective learning behaviours can promote students’ learning outcomes in the museum. Using strategies such as museum scaffolding, voice guided learning, video guided learning, sub museum sequencing, points on the list and video guided learning, the intervention study was conducted on the museum learners who needed the intervention. The results of the intervention showed that the learning status of some learners improved significantly, and from Figs 10 and 11, we calculated that 89.3% of the stray type of museum learners had moved out of the stray state after the intervention and The other three types of learners were transformed. The teacher of learning in the museum can make use of the continuous, real-time, cyclical cluster analysis and identification modelling of learner behaviour data to draw a picture of the group of learners at the beginning of learning in the museum, to identify patterns of learning behaviour, and to identify learners who need intervention as soon as possible, so as to facilitate the improvement of learning outcomes.

## VIII. Conclusions

This study conducted an experimental research to explore how to solve the wandering phenomenon that learners are prone to in virtual museums, and how to conduct museum teaching interventions. The 2030 learners of clinical medicine in a university in Northeast China were selected, and the behavioral data collected in the background when the learners visited the virtual museum was used as the data source, and methods such as cluster analysis of fuzzy C were used to examine the results based on the identification of learning behaviors in the virtual museum, while the sequence transformation of learning behaviors in the virtual museum was carried out based on the lagged sequence analysis method, so as to obtain four types of learners: high immersion type in the museum, medium immersion type in the museum, low immersion type in the museum and stray type in the museum. The high immersion learners are more self-regulated and do not need to participate in subsequent intervention experiments. In this study, the four categories of learners were divided into two types of learners: medium-high immersion and low-loafing, and the importance of their behaviour was identified through the random forest algorithm. The results showed that the learning status of some learners improved significantly, especially the stray learners got more than 80% improvement, which is important to improve the overall field learner effectiveness of students and lay the foundation for future research on deep learning in the field. Teachers should identify learning behavior patterns as early as possible to determine corresponding interventions, so as to improve their learning effect. The builders of virtual museums should provide learners with personalized learning experience by combining the characteristics of both virtual and brick-and-mortar museums.

This study also has some limitations. Due to the pandemic of COVID-19, cycle experiment can not be carried out. The scope and quantity of experimental subjects are affected by many factors. Specifically, this experiment was only performed in a university in Northeast China and the data of students’ learning within 30 minutes were used for analysis and research. In future study, the experimental research areas and sample number should be expanded. Moreover, the state of learners also changed with the passage of time, so the follow-up study of our experimental team will perform several cycle experiments according to learners’ learning cycles with the aim to explore whether the passage of time and the enhancement of learners’ relevant knowledge reserve can influence learning effect [28]. In this study, sex was not included as one of the interference factors, so future study should further investigate and verify whether sex influences virtual museum experiments. In terms of the function of learning behavior recognition, only the museum backstage supporter recorded learners’ learning behaviors and in future virtual museum learning, such techniques as eye movement experiment [29], electroencephalogram (EEG) experiment [30], emotion monitoring [31] and others will be introduced to assist with the collection of multimodal data. In museum learning, in-depth future research on the improvement of learners’ learning effect, the evaluation model of learning effect, the design and development of museum teaching intervention system and others are required.

Virtual museums boast high immersion and are not limited by time and space [32], so our experimental team will continue to apply virtual museums to university teaching in the future, so as to provide college students with the best virtual learning environment and learning resources.
